# *Escherichia coli* pathobionts and Crohn’s disease: varied genetic paths leading to similar phenotypes

**DOI:** 10.1128/jb.00016-26

**Published:** 2026-04-30

**Authors:** Melissa Arroyo-Mendoza, Hernan Lorenzi, Gregory J. Phillips, Deborah M. Hinton

**Affiliations:** 1 Gene Expression and Regulation Section, Laboratory of Biochemistry and Genetics, National Institute of Diabetes and Digestive and Kidney Diseases, National Institutes of Health 2511, Bethesda, Maryland, USA; 2 TriLab Bioinformatics Group, National Institute of Diabetes and Digestive and Kidney Diseases, National Institutes of Health 2511, Bethesda, Maryland, USA; 3 Department of Infectious Diseases, University of Georgia 1355, Athens, Georgia, USA; University of Virginia School of Medicine, Charlottesville, Virginia, USA

**Keywords:** RNA polymerase, *E. coli* pathobiont, LF82, Crohn's disease

## Abstract

Crohn’s disease (CD), an inflammatory bowel disease that arises from an immune attack on the gastrointestinal tract, affects roughly 1.6 million Americans. The etiology of CD and the other major irritable bowel disease, ulcerative colitis, is not known, but host genetics and immunology, the gut microbiome, and environmental factors are all thought to be involved. In addition, adherent-invasive *Escherichia coli* (AIEC) strains, which are able to adhere to and invade epithelial cells and macrophages, are frequently found to be associated with CD. Besides their adherence and invasion properties, key features that distinguish AIEC from commensal *E. coli* include increased biofilm formation, increased antibiotic resistance, and survival/replication within macrophages. However, these pathobionts lack genetic features typical of frank pathogens. Thus, the potential role AIEC plays in CD pathogenesis is not clear. The *E. coli* pathobiont LF82, isolated from the ileum of a patient with CD, has been a well-studied, prototypic AIEC. Dozens of single-nucleotide polymorphisms (SNPs) distinguish LF82 and other AIEC from commensal *E. coli*, suggesting that some of these genetic features might account for particular LF82 phenotypes. In this review, we summarize changes in the CD gut, the association of AIEC with CD, genes and SNPs associated with AIEC, and recent work connecting a specific SNP within a bacterial RNA polymerase gene to the expression of genes associated with the LF82 lifestyle.

## INTRODUCTION

With approximately 2.4 million cases of irritable bowel disease (IBD) in the United States (1), IBD is one of the top five most expensive GI conditions, causing a financial burden in the U.S. of ~ $50 billion annually (2). The two major clinical forms of IBD are ulcerative colitis (UC) and Crohn’s disease (CD). Both involve chronic and recurring inflammation that leads to damage of the gastrointestinal (GI) tract and severe GI symptoms, including abdominal pain, diarrhea, bleeding, weight loss, anemia, and extraintestinal symptoms (3, 4).

UC and CD appear to be “immune dysregulation diseases,” in which the underlying issue is an inappropriate ongoing activation of the mucosal immune system in response to antigens from gut bacteria (5, 6). Host genetics and immunology, the gut microbiome, and environmental factors all appear to contribute to disease progression (6–8). While the precise etiology of CD and UC is not understood, the increased presence of adherent-invasive *Escherichia coli* (AIEC) in the CD and UC gut suggests a potential role for these bacteria. In this review, we discuss the association of AIEC pathobionts with CD and recent work that elucidates how a single-nucleotide polymorphism (SNP) within the AIEC LF82 may contribute to the pathobiont’s lifestyle.

## UC AND CD HISTORY AND LOCALIZATION

The first description of UC is attributed to Hippocrates of ancient Greece, but the actual term “ulcerative colitis” was coined in 1859 by British physician Sir Samuel Wilks (9). CD was named after gastroenterologist Dr. Burrill Crohn, who, in 1932, along with colleagues Drs. Leon Ginzburg and Gordon Oppenheimer published an article describing patients with chronic inflammation of the terminal ileum as well as transmural inflammation, strictures, and fistulas (10).

Both UC and CD involve gut inflammation, but they are differentiated by their clinical manifestations of intestinal localization and inflammation (11). UC is a disease of the colon with chronic uncontrolled mucosal inflammation extending from the rectum to the proximal colon segments. In contrast, CD most commonly affects the terminal ileum and the colon but can involve any part of the GI tract. Both UC and CD can lead to chronic, long-term disease with severe complications such as anemia, osteoporosis, and cancer (12–14). For UC, megacolon is a rare but life-threatening complication (15). For CD, surgical operations, bowel damage, and disability are frequent outcomes, and about half of the CD patients who present the inflammatory-like symptoms will eventually develop complications such as strictures, fistulas, or abscesses (16).

## POSSIBLE ETIOLOGIES OF CD

### Immuno-dysregulation

From the 1970s until about two decades ago, the investigation of IBD pathogenesis primarily focused on the study of immunological processes since IBD is associated with inflammation within the gastrointestinal tract (17, 18). The inflammation arises from an aberrant mucosal immune response, specifically a dysregulation of the innate and adaptive immune systems in response to signals from damaged tissue and the intestinal microbiota (16). Production of inflammatory cytokines and factors by the innate immune response stimulates T-lymphocytes (T cells) and B-lymphocytes, the key cells of the adaptive immune system, generating a repetitive cycle of inflammation and host immunological response (19).

**Fig 1:**
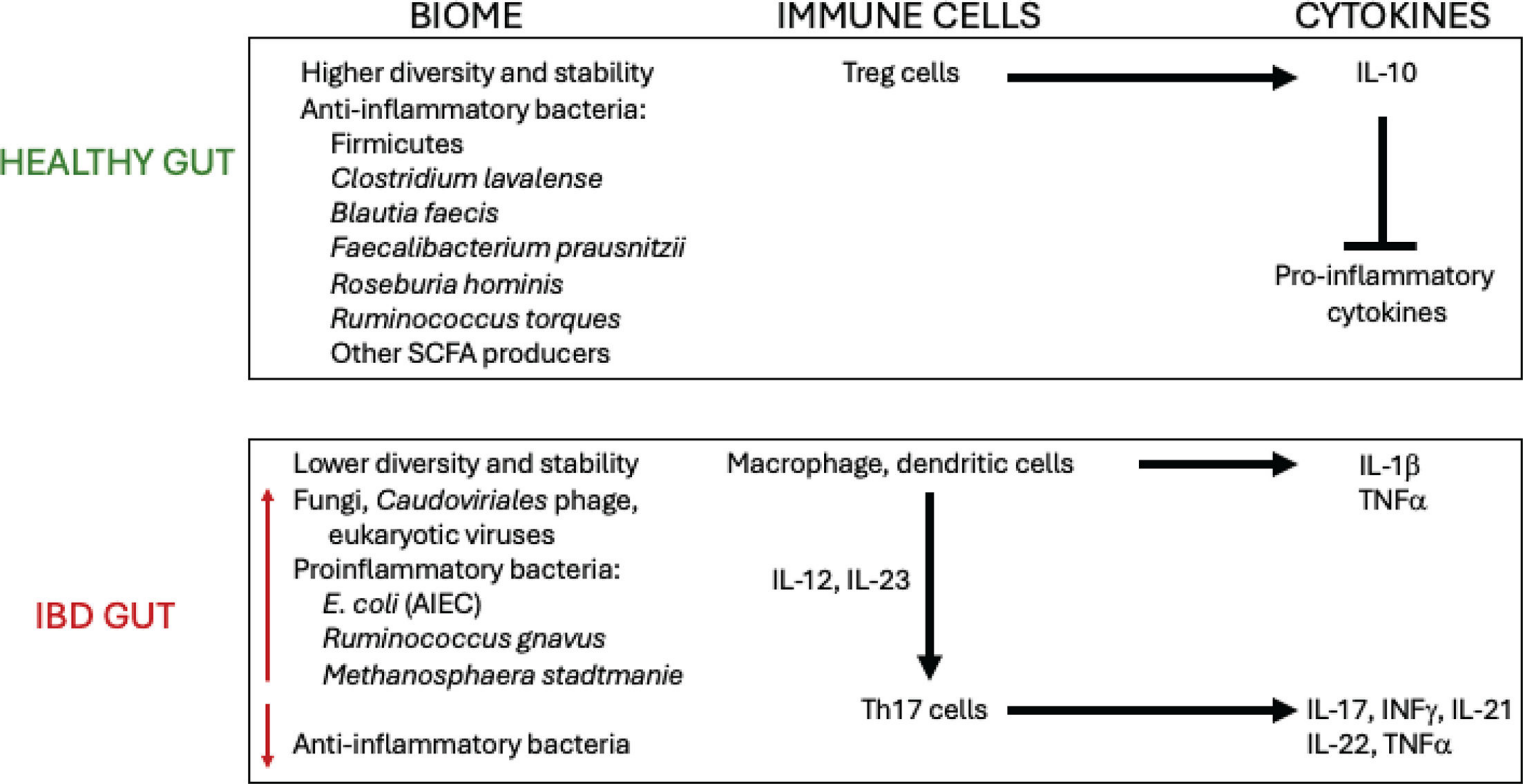
Overview of the gut composition in a healthy vs*.* IBD gut. Adapted from Núñez-Sánchez et al., 2022 (27).

Most T cells are composed of CD4^+^ and CD8^+^ subtypes. Upon activation, the CD4^+^ T cells differentiate into distinct effector subtypes that include Th1, Th2, Th17, and regulatory T cells (Tregs) (Fig. 1). Each of these subtypes plays a unique role in pathogen elimination and contributes to mediating the immune response through the secretion of specific cytokines (20, 21). In CD, immuno-dysregulation arises from the accumulation of activated CD4^+^ T cells in the intestinal tissue and the buildup of their associated proinflammatory cytokines (19). CD was originally classified as a Th1-mediated disease. This classification arose in part from (i) the increased level of the Th1-associated transcription factor T-bet in the lamina propria T cells from CD patients (22); (ii) altered levels of STAT4 isoforms mRNAs, which encode another Th1-associated transcription factor, in cells from biopsies of CD patients (23); and (iii) the production of the Th1-associated cytokines IL-12 (24) and INF-γ (25, 26) in lamina propria from CD patients.

Other work has also implicated Th17 cells that secrete proinflammatory cytokines IL-17, IL-21, IL-22, and TNFα, which are involved in host defense at the mucosal surface (Fig. 1) (28, 29). While the anti-microbial properties of these proinflammatory mediators are helpful during infection, in CD, the Th17 response contributes to disease progression by promoting inflammation (30).

### Genetic susceptibility

More than 200 human alleles have been associated with IBD, with 41 specific for CD (6). Some of these genes are involved with innate immunity, bacterial sensing, Th17 cell function, or an altered mucus layer (16). A large fraction of the heritable risk for CD can be explained by mutations in three genes: *NOD2* (the intracellular pattern recognition receptor that recognizes pathogens), *ATG16L1* (involved in cell autophagy), and *IL23R* (the IL-23 receptor) (31). However, only about 12% of CD patients have a family history of the disease, and only around 13% of disease heritability can be explained by genetic variation (16).

### Environmental risk factors

Smoking, diet, drugs, stress, geography, social status, and the gut microbiota are all environmental factors that modify effector function of the intestinal immune system and alter the expression of genes in susceptible individuals (6, 16, 32). Smoking promotes a fistulizing and structuring phenotype, worsens clinical course, and is associated with suboptimal response to medical therapy in CD (33). Ironically, smoking appears to be protective for UC (34). The use of nonsteroidal anti-inflammatory drugs and oral contraceptives has also been correlated with increased CD risk (35, 36).

The use of antibiotics in early life stages is associated with an increased risk of IBD as it can disrupt the intestinal microbiome and therefore affect infant immune development (37, 38). It is thought that during early life stages, the microbiota play a critical role in shaping the immune cell development.

Given the location of CD, it is only logical that there is a likely association between diet and disease. Westernized diets, which generally consist of a low intake of fiber and vegetables with a high intake of saturated fats, red meat, carbohydrates, polyunsaturated fats, and sugar, are associated with higher rates of CD (39). Previously low-risk Asian countries are now experiencing a sharp increase in the incidence of IBD with urbanization/industrialization and adoption of a western lifestyle, including diet (40). It is thought that diet can negatively impact microbial diversity by altering the intestinal microbial composition and function.

## GUT DYSBIOSIS PRESENT IN CD

One of the key features of CD is microbial gut dysbiosis, i.e., alterations in the structure and function of the gut microbiota (8, 27, 41). In addition to changes in bacterial abundance, dysbiosis also includes changes in fungi, bacteriophage, and archaea (42). Figure 1 summarizes hallmark attributes of a healthy vs*.* IBD gut. In particular, the abundance of bacteria that produce short-chain fatty acids (SCFAs), such as *Clostridium*, *Faecalibacterium prausnitzii*, *Eubacterium*, and *Roseburia hominis*, is lower in the CD gut than in a healthy gut (8, 27). This decrease is detrimental as SCFAs help maintain the integrity of the gut barrier and assist in the differentiation of anti-inflammatory T cells (43, 44). Furthermore, there is an increase of *Ruminococcus gnavus* and *Methanospaera stadtmaniae*, which promote the release of proinflammatory cytokines such as TNFα (45–47). The level of the opportunistic fungal pathogen *Candida albicans* also increases. This can be problematic as *C. albicans* resides in the gastrointestinal tract and interacts with the Th17 cells, leading to inflammation (47). Finally, the increased presence of pathobionts, such as AIEC, is associated with the CD gut (48, 49).

## AIEC PATHOBIONTS AND PHENOTYPES

Pathobionts are microbes that can be found within the commensal microbiota but can also display pathogenic properties when given the correct opportunity, context, and circumstance (50). As neither a true commensal nor a frank pathogen, AIEC has distinctive characteristics that differ from those of *E. coli* pathogens, such as enterotoxigenic, enteropathogenic, and uropathogenic *E. coli* and from commensal *E. coli* strains and common lab strains, such as MG1655 (51). A major distinguishing characteristic of AIEC is the ability to adhere, invade, survive, and replicate within epithelial cells and macrophages with the accompanying induction of TNFα secretion, even though AIEC lacks typical virulence factors (52).

As a pathobiont, AIEC is found in both CD and healthy individuals, but it is found in 51.9% of CD patients, while only in 16.7% of normal controls (53). It is thought that the inflammatory environment of the CD gut, which can be detrimental for commensal microbes, provides a selective advantage for other microbes, such as AIEC and other proteobacteria (54). These microbes then contribute to and help sustain the inflammatory response, dampening the recovery of the commensal microbes.

## GENOTYPES OF AIEC

Understanding the genetic differences of AIEC that give rise to its distinctive phenotypes has been a top priority of IBD research. Dozens of AIEC genomes, such as LF82, NRG857c, HM605, and UM146, have been partially or completely sequenced (55–60). *E. coli* LF82 (for Lille-France), isolated from the ileum of a patient with CD in 1998 (61), was one of the first identified AIEC, and both LF82 and *E. coli* NRG857C are considered prototypic AIEC strains. They both belong to the O83:H1 serotype and are genetically very similar (62).

**Fig 2:**
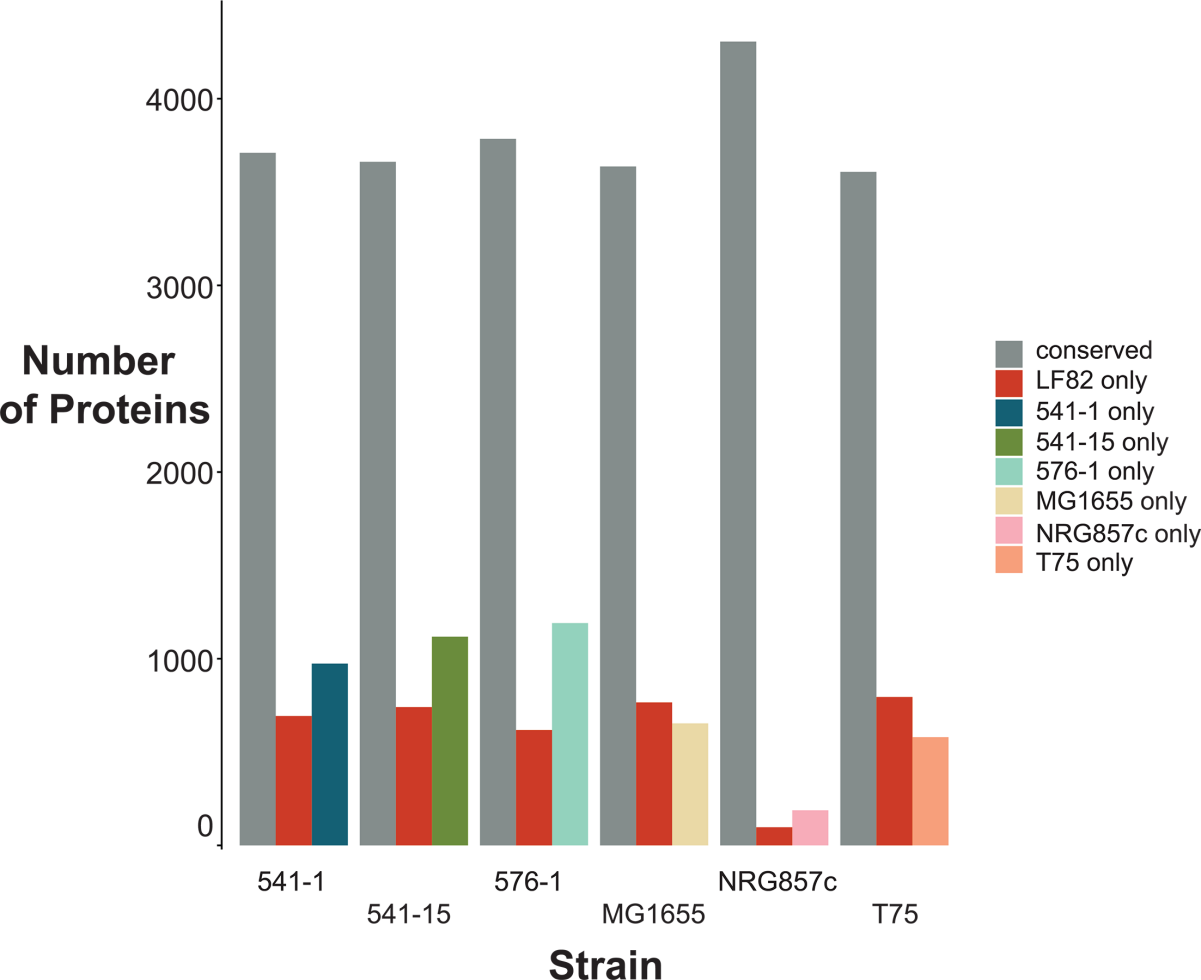
Quantification of conserved protein-coding genes between *Escherichia coli* LF82 and six other *E. coli* strains. Protein sequences from each strain were compared to those of LF82 using BLASTp (v2.15.0+). Conserved proteins were defined as best reciprocal matches (BRM) with an *e*-value ≤ 1 × 10⁻¹⁰, percent identity ≥35%, and alignment coverage ≥50%. Proteins lacking reciprocal hits or failing to meet these thresholds were classified as strain-specific in each pairwise comparison. The code for performing this analysis can be found at https://doi.org/10.5281/zenodo.17992151.

Sequencing of AIEC genomes has revealed various SNPs among AIEC, commensal *E. coli*, and *E. coli* lab strains. From these analyses, genes have been identified that might be involved in the hallmark characteristics of adhering, invading, and replicating in macrophages, surviving in host cells, and inducing proinflammatory cytokine secretion. However, the various AIEC isolates do not carry a signature genetic make-up and do not even belong to the same phylogroup within the *E. coli* species. While most AIEC strains belong to the B2 subgroup *of E. coli*, strains have also been identified as A, B1, and D (52, 59, 63). Furthermore, multiple genes related to AIEC “pathogenicity” such as *fimH*, *ompA*, *dsbA*, and *htrA* are not AIEC-specific and can be found in many *E. coli* strains, including non-pathogenic ones (62). A comparison of the LF82 encoded proteins to those of the *E. coli* lab strain MG1655 and to those of other AIEC (541-1, phylogroup B1; 541-15, phylogroup A; 576-1, phylogroup D; T75, phylogroup A; NRG857c, phylogroup B2) reveals large differences (Fig. 2). As expected, LF82 and NRG857c are by far the most similar, but LF82 shares as much similarity with MG1655 as with the other AIECs. This phylogenetic distribution suggests that AIEC phenotypes have evolved independently by various pathways as they have adapted to specific environmental pressures/conditions. Consequently, the characteristic AIEC phenotypes are thought to have arisen through different combinations of genes instead of a particular set of genes, and there must be several genetic paths that can lead to characteristics needed for success in the CD gut. Because of the lack of specific molecular biomarkers, the only way to identify an AIEC isolate has been from its phenotypic traits, i.e., assessing phenotypes and interactions between host and bacterial cells (64).

## MOUSE MODELS FOR INVESTIGATING CD

The binding site for AIEC is the CEACAM6 (carcinoembryonic antigen-related cell adhesion molecule 6) receptor on the surface of human ileal epithelial cells (48), which is bound by the *E. coli* Type I pili. However, the mouse gut lacks this receptor, making it a less-than-ideal model for CD. Nonetheless, under certain conditions, the AIEC properties of adherence and invasion, the establishment of inflammation, and a CD-like immune response can be observed after introduction of AIEC into various standard lab mouse strains (65–67). In these cases, mice are typically treated with antibiotics and/or dextran sodium sulfate salt (DSS), a compound that induces inflammation, to facilitate the process. More recently, a transgenic mouse model containing the CEACAM6 receptor has been established, allowing one to investigate the AIEC–CEACAM6 interaction *in vivo* (68, 69). While streptomycin-treated conventional mice have been chronically maintained with AIEC NRG857 for months with various traits symptomatic of CD pathology (70), the introduction of LF82 into any of these models does not result in long-term colonization and maintenance, as is seen in human CD. In fact, the presence of LF82 normally kills most of the mice within days to a few weeks.

Because of these issues, the Altered Schaedler flora (ASF) gnotobiotic mouse model has also been employed since it allows long-term, generational colonization of *E. coli* LF82 (71) as well as other bacteria involved in chronic diseases, including *Helicobacter bilis* and *Campylobacter jejuni* (72, 73). In the ASF gut, there are only eight bacterial species, and none is a member of the *Enterobacteriaceae* (72). Because of this and in contrast to conventionally reared mice with a complex microbiota, ASF mice stably maintain LF82 for multiple generations without the need for continual reacquisition or disruptive antibiotic treatment (71).

## GENES AND SNPs IN AIEC THAT HAVE BEEN ASSOCIATED WITH CD

Given the similar phenotypes of AIEC, it has seemed likely that specific genes or SNPs might be involved in the AIEC lifestyle, even though no common genetic loci have been found. Several recent transcriptomic and genomic mutational investigations have focused on undercovering these possible commonalities.

One gene found in AIEC, including LF82 and NRG857C, but not in nonpathogenic *E. coli*, is *ibeA* (invasion of the brain endothelium protein A), which is involved in newborn meningitis caused by an *E. coli* Neonatal Meningitis-causing *E. coli* (NMEC) isolate (74). A role for IbeA has also been implicated in CD. Deletion of *ibeA* in NRG857C lowers its ability to invade intestinal epithelial cells and to survive in human macrophages (75). In addition, recent work has found that IbeA is a flavin adenine dinucleotide binding protein and suggests that it may function to modulate the acidic environment of the macrophage, increasing survival within the macrophage (76).

Transcriptomics studies comparing the gene expression of LF82 to nonpathogenic *E. coli* have identified various genes that are upregulated in the AIEC, suggesting that they may be involved in CD. These include genes related to bacteriophage infection, inorganic ion transport, chemotaxis/motility, and metabolism (77), and the *eut* operon involved in ethanolamine utilization (78). The level of ethanolamine increases in the gut during periods of inflammation (79), and the growth of LF82 within macrophages has been shown to increase with an increase in ethanolamine concentration (80).

Transcriptomics analyses have also been performed by focusing on gene expression within intestinal cells or in the mouse. A study investigating gene expression differences inside intestinal cells using AIEC and non-AIEC pairs derived from two patients (81) revealed 37 differentially expressed genes. The eight overexpressed genes (*fimC*, *fimD*, *fimF*, *pstA*, *pstC*, *sufE*, *yjjB*, and *yjjP*) implicated functions involved in phosphate transport, iron-sulfur cluster assembly, succinate export, and in the synthesis of the Type I pili that are needed for attachment to CEACAM6 (48). Underexpressed genes included those involved in colonic acid synthesis, anaerobic respiration, and tRNAs.

Since the AIEC strain NRG857C can be maintained in mice, a combination of transcriptomics and transposon insertional mutations has been performed, investigating the expression and phenotypes needed for this strain to colonize the mouse intestine (82). This work revealed the importance of AIEC to express genes involved in nitrate metabolism; two-component systems; amino acid transport and metabolism; fucose, galactitol, and ethanolamine utilization; iron acquisition; and the type four secretion system (T4SS). The T4SS was crucial for intestinal colonization and for the formation of biofilm on intestinal cells, presumably a protective mechanism within the gut.

Genes associated with propanediol utilization (*pdu* operon) and iron and heme acquisition (*chu* operon) have been found more frequently in AIEC than in nonpathogenic *E. coli*, and their presence correlates with increased cell invasion (63). A SNP within the *blc* gene, which encodes an outer membrane lipoprotein involved in lipid storage/transport, has been identified in *E. coli* strains that are enriched in the feces of IBD patients, including LF82 and NRG857C (83). Introduction of *E. coli* that produces the Blc variant, in which the glycine at residue 84 has been converted to glutamic acid, results in epithelial barrier disruption and immune activation in mice, suggesting that the *blc* SNP may facilitate or exacerbate IBD.

Because LF82 can be stably maintained in the ASF mouse, it has been possible to examine genetic changes that arise in the LF82 genome as LF82 is passed from one generation of mice to the next. In this experiment, the mice were treated with DSS to induce inflammation or with water as a control. However, most of the genomic changes were seen without DSS. This work revealed that >400 new SNPs were generated as LF82 passed through five generations of mice (71). The vast majority of these changes appeared to be genetic drift. However, in three cases, the change strikingly “reverted” a rare substitution that was present in WT LF82 (before passage) back to the highly conserved sequence found in most *E. coli*, including MG1655 (Table 1). Interestingly, two of the reversions occurred in genes encoding subunits of RNA polymerase (RNAP), the enzyme that is essential for gene expression since it transcribes genomic DNA into RNA. This result has suggested that in ASF mice, the long-term maintenance of LF82 may be associated with a return to a more “commensal-like” state for RNAP. The third gene was *rpiB*, which encodes an allose-induced ribose phosphate isomerase that functions within a pathway degrading D-allose (84, 85). Recent work using a mouse model of colitis-associated carcinogenesis found that the administration of D-allose reduced both inflammation and tumor count, suggesting that D-allose may have a role in IBD (86). However, the effect of a valine to alanine substitution at residue 59 of RpiB has not been determined. In contrast, the *rpoD* change within RNAP has suggested that LF82 might take advantage of a SNP within the highly conserved bacterial transcription machinery to alter its genotype/phenotype.

**TABLE 1:** Substitutions present in the LF82 RpoD, RpoB, and RpiB proteins that “revert” after passage through ASF mice

Difference between LF82 and MG1655^*a*^				
Protein	Position^*b*^	LF82 residue	MG1655 residue	Reverts after passage?^*c*^
RpoD	205	Threonine (T)	Serine (S)	No
	229	Isoleucine (I)	Valine (V)	No
	244	Alanine (A)	Threonine (T)	No
	**445**	**Valine (V)**	**Aspartic acid (D)**	**YES**
	571	Histidine (H)	Tyrosine (Y)	No
RpoB	**1263**	**Glutamic acid (E)**	**Alanine (A)**	**YES**
RpiB	**59**	**Alanine (A)**	**Valine (V)**	**YES**

^*a*^ Residues that differ between the indicated encoded proteins in WT LF82 (before passage through ASF mice) and WT MG1655.

^*b*^ Residue position within protein.

^*c*^ Indicates “yes” and line is shown in bold if the substitution in LF82 “reverts” to the MG1655 residue after passage in ASF mice.

## DIFFERENCES BETWEEN RNAP GENES IN COMMENSAL MG1655 AND THE AIEC PATHOBIONT LF82

**Fig 3:**
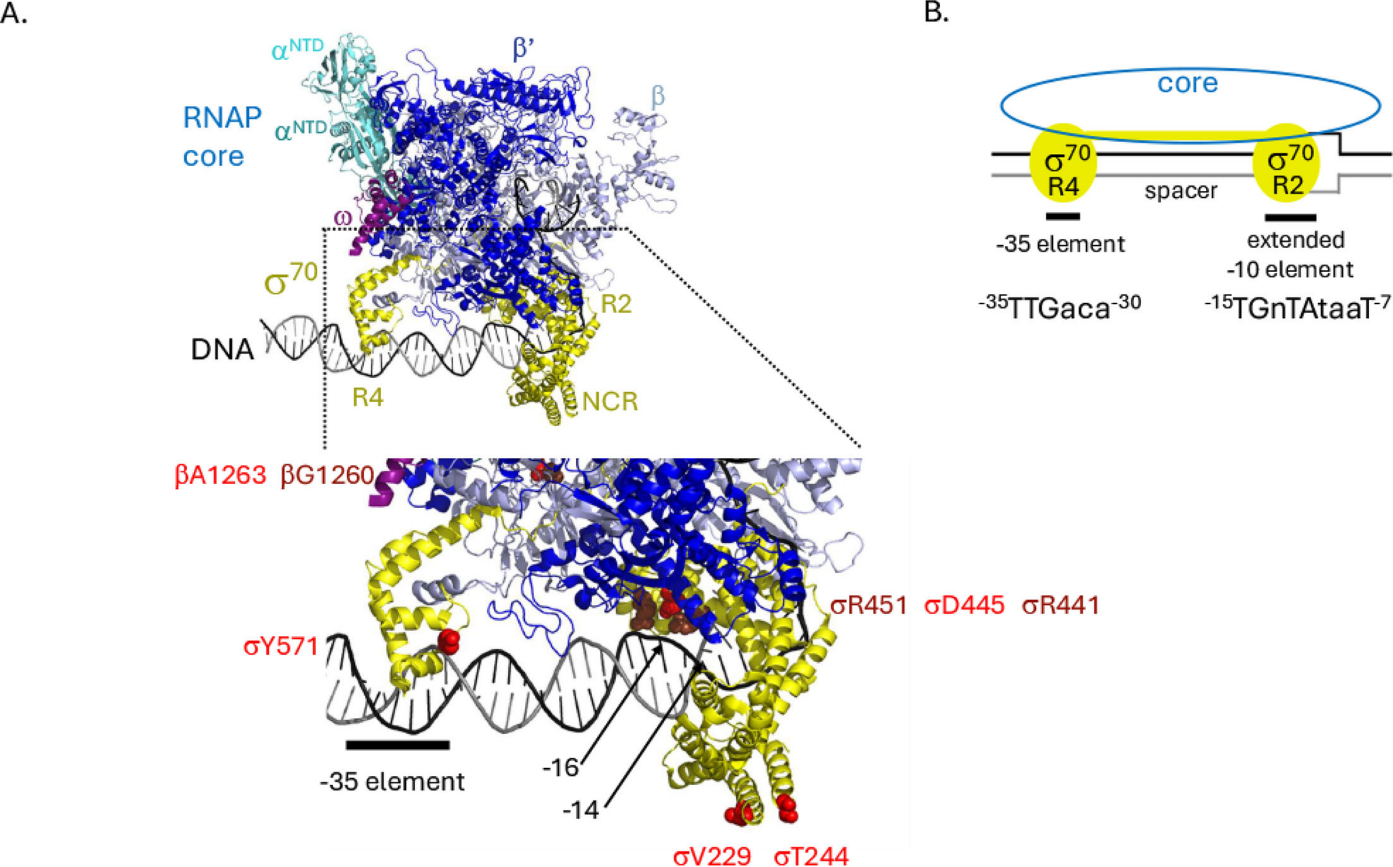
Positions of substitutions within the AIEC strain LF82 σ^70^-RNAP relative to the *E. coli* lab strain MG1655 σ^70^-RNAP. (**A**) Top shows the structure of the *E. coli* RNAP/promoter DNA complex (PDB:6CA0 [91]). The core subunits (β, light blue; β’, dark blue; N-terminal domains of the 2 α subunits [α^NTD^s], teal; and ω, magenta), σ^70^ (yellow), and DNA (top strand in black, bottom strand in dark gray) are shown. The σ^70^ regions, R2, R4, and the non-conserved region (NCR), are indicated. Underneath is an expansion of the indicated portion of RNAP showing the residues (as red spheres) where there are differences between MG1655 RNAP and that of LF82: β A1263, σ^70^ Y571, σ^70^ D445, σ^70^ T244, and σ^70^ V229. (The other σ^70^ variant at S205 is not visible in the structure but is also located in the NCR.) β G1260, which is within 5 Å of A1263, and σ^70^ R441 and σ^70^ R451, which are within 5 Å of σ^70^ D445, are shown as dark brown spheres. The location of the −35 element and positions −16 and −14 on the top strand are indicated. (**B**) Cartoon representation of RNAP in (A) showing core in blue and σ^70^ R2 and σ^70^ R4 in yellow relative to the DNA (black/gray) with the positions of the −35 element and the extended −10 element indicated. The top strand 5′→ 3′ consensus sequences are shown with lowercase denoting a less conserved sequence and “n” representing any base; positions are relative to the transcription start site at position +1 within an ideal promoter. The transcription bubble present at the start of transcription from −11 to ~+3 is indicated.

RNAP is an essential enzyme for all organisms, providing the primary control for gene expression. Consequently, RNAP is conserved throughout biology. In bacteria, RNAPs contain an RNA-synthesizing core, consisting of two large subunits [β (*rpoB*) and β′ (*rpoC*)], two α (*rpoA*) subunits, and one ω (*rpoZ*) subunit, together with a σ specificity factor that is needed to recognize a DNA promoter sequence and set the start site for transcription (87) (Fig. 3A). The *rpoD* gene encodes the primary and essential σ factor, called σ^70^ in *E. coli*. It directs RNAP to transcribe “housekeeping” genes that are required for exponential growth and is kept at a constant level even during the stationary phase (88, 89). Bacteria typically also encode alternative σ factors, which allow RNAP to transcribe genes needed for growth under specific conditions and at times of stress (90).

In the context of RNAP, highly conserved regions of σ^70^ recognize two primary promoter DNA sequences, σ^70^ Regions 2.4/2.3 with the extended −10 element and σ^70^ Region 4.2 with the −35 element (92) (Fig. 3B). The sequence between promoter positions −30 and −12 is known as the spacer; a spacer length of 17 is ideal, but a 16 or 18 bp spacer is allowed. Except for the ^−15^TG^−14^, the spacer is thought to have little sequence information, although residues within σ^70^ Region 2.4 are in contact with the downstream portion of the spacer DNA.

In LF82, there are five substitutions within σ^70^ that differ from those in σ^70^ in MG1655 (Table 1). Three substitutions are found within the σ^70^ non-conserved region (NCR), which, as the name implies, is a much less conserved portion of σ^70^. The fourth substitution is a conservative change at the beginning of σ^70^ Region 4.2 (not near DNA or other subunits). However, the fifth substitution and the one that reverts after passage in ASF mice, D445V, imparts a charge change and is located within a very highly conserved portion of Region 2.4. More than 97% of the primary σ’s found in >20,000 *Enterobacteriales* genomes have an aspartic acid at residue 445, whereas 0.01% have a valine. Besides LF82, two additional *E. coli* genomes (HCO1463624.1 and HAM6750629.1) have the D→V substitution. Interestingly, the D→V substitution is also not found in other AIEC (57, 58, 77, 93–97).

The LF82 substitution found in the β subunit of RNAP is β A1263E, which also “reverts” after passage through ASF mice. Residue 1263 is located within core RNAP, in the highly conserved β Region I, a C-terminal domain of the β subunit (98, 99). Although there is no information about substitutions at A1263, the residue is within 5 Å of G1260 (91), where a G→E substitution has been shown to decrease the half-life of an RNAP/promoter complex, to decrease the intrinsic RNA cleavage of RNAP, and to suppress transcription-replication conflicts under certain conditions (100).

It was particularly surprising to find the LF82 SNPs for σ^70^ D445 and β A1263 since these substitutions result in significant charge changes in highly conserved regions of RNAP. The conservation of these residues and their positions within RNAP implies that these mutations might have important consequences for LF82 gene expression.

## INVESTIGATION OF RpoD D445V IDENTIFIES A LF82 SNP RELATED TO THE AIEC LIFESTYLE

To investigate whether the RpoD D445V substitution affects LF82 gene expression, phenotypes, and transcriptomes of MG1655 WT vs*.* D445V and LF82 WT vs*.* V445D have been compared; this work indicates that the presence of σ^70^ V445 in either background results in 24 shared, upregulated genes (71). Importantly, as detailed below, several of the shared genes are consistent with LF82 phenotypes.

### metE

MetE catalyzes the final step in methionine biosynthesis in the absence of vitamin B12 (101). The presence of σ^70^ V445 improves the ability of MG1655 to grow in the absence of exogenous methionine, suggesting that it is better able to synthesize the amount of methionine it needs for optimal growth (71). Other work has found an increase in methionine and methionine metabolites in fecal material from CD patients (102), which is consistent with an increase in methionine levels and correlates with the ability of LF82 to increase methionine production.

### fucI

*fucI* encodes L-fucose isomerase needed for the first step in fucose degradation (103). Fucose is found in the gut as fucosylated glycans and fucosyl oligosaccharides, which are then processed by the gut microbiota to generate the beneficial SCFs (104). In addition, administration of L-fucose has anti-inflammatory effects in mouse colitis models (105). Consequently, an increase in *fucI*, which should lead to the degradation of fucose, is counterproductive for the healthy gut.

### blc

Blc is an outer membrane lipoprotein (106). As discussed earlier, previous work has shown that a Blc variant (G84E), which is present in dozens of pathogenic *E. coli* strains and in the AIEC strains LF82 and NRG857C, but not in commensal strains, leads to epithelial barrier disruption and immune activation in the mouse (83). This suggests that upregulation of the Blc variant by the RpoD variant would be associated with an increase in gut inflammation.

### yobH

Although the function of YobH in *E. coli* has not been determined, in *Salmonella*, *yobH* is required for macrophage invasion (107). This suggests that it may play a similar role for macrophage invasion by LF82.

### Genes involved in antibiotic resistance

Antibiotic resistance is a phenotypic hallmark of AIEC strains. For example, LF82 is known to be ampicillin resistant (amp^R^) and to have an antibiotic resistance pattern that differs from that of other *E. coli* (108). The presence of σ^70^ V445 increases the level of the *ampC* RNA, encoding β-lactamase, and increases resistance to ampicillin and other β-lactam antibiotics in either the MG1566 or LF82 background (71). Other genes involved with multidrug transporters *acrZ* and *bcr* are also upregulated by the presence of σ^70^ V445 with accompanying alterations in antibiotic resistance.

The possible importance of the shared, upregulated genes for LF82 biology has also been inferred from comparing the 24 genes whose expression increases with σ^70^ V445 in both the MG1655 and LF82 backgrounds to a list of LF82 genes whose expression significantly changed after macrophage invasion (109). This analysis indicated that the expression of most of the 24 genes was altered after macrophage invasion. In particular, the levels of *yobH* and *fucI* increased after invasion. However, it is important to note that the levels of hundreds of LF82 genes changed post-invasion, suggesting, as expected, complex patterns of gene expression changes.

In contrast to the many shared upregulated genes, only four downregulated genes were common between the MG1655 and LF82 backgrounds: *gltP* (glutamate/aspartate transporter), *folD* (methylenetetrahydrofolate dehydrogenase), *ruvA* (DNA binding protein for Holliday structures), and *yacC* (putative lipoprotein). How the downregulation of these genes may be related to LF82 function is not clear.

## THE σ^70^ D445V SUBSTITUTION INCREASES TRANSCRIPTION INITIATION AT SPECIFIC PROMOTERS

To test whether RNAP σ^70^ V445 directly affects transcription, various DNA templates were transcribed in single round *in vitro* transcription assays using RNAP containing σ^70^ D445 or σ^70^ V445 (71). Tested promoters included ones found upstream of genes in the shared, upregulated data set (P*_ampC_*, P*_yobH_*, P*_lptM_*, P*_yciY_*, P*_xseA_*, P*_csrD_*, and P*_sspA_*) as well as others upstream of unaffected genes. These analyses demonstrated that at the upregulated genes, transcription increased approximately twofold when using σ^70^ V445, indicating that this single SNP can effectively change gene expression at specific genes by directly increasing transcription initiation.

Curiously, among the 14 identified promoters for the shared, upregulated genes, 9 have a 16 bp spacer (1 bp shorter than the ideal), 10 have a −14 G, and 8 have both features (57%). However, the 16 bp spacer/^−14^G combination is found in only ~5% of the hundreds of known *E. coli* promoters (110, 111). This suggested that the σ^70^ V445 variant increases promoter activity based on the sequence and/or structure of the spacer region in these promoters. Transcriptions using *P_ampC_* with targeted mutations confirmed that for *P_ampC_*, it is the 16 bp spacer length that determines the preference for σ^70^ V445 (71).

How can the spacer of a specific promoter increase transcription by σ^70^ V445-RNAP? The promoter DNA just downstream of the spacer must bend dramatically as it interacts with RNAP, and the angle of this bend establishes the position of the −10 element for this interaction (91, 112). Importantly, the length and the sequence of a spacer influence the angle of the bend (113–115). Structures of RNAP with promoter DNA (91, 112) indicate that two σ^70^ arginines, R441 and R451, are in close contact with σ^70^ D445, but they are also in close contact with the spacer DNA at the positions −14 and −16, respectively (Fig. 3A). Consequently, depending on the angle of the bend, a glutamic acid at residue 445, the typical residue for most *E. coli*, may compete with the negatively charged spacer bps for interactions with these arginines. This will then decrease the interaction of RNAP with the promoter. However, when the nonpolar valine is present at 445, as it is in LF82, it cannot compete, and the contact between R441/R451 and the DNA will be stronger, increasing the overall RNAP/DNA interaction. Thus, the combination of a specific spacer length, sequence, and σ^70^ V445 can result in a more active promoter. However, since not every *E. coli* 16 bp spacer promoter or ^−14^G promoter is affected by σ^70^ V445, the overall sequence/structure of the promoter and the rate-limiting step for transcription initiation must also influence whether the σ^70^ V445 substitution will have an effect.

## THE TRANSCRIPTOMIC EFFECT OF THE RpoB A1262E SUBSTITUTION APPEARS TO BE MINIMAL

To investigate the effect of having a glutamic acid at β1263, which is found in WT LF82, instead of an alanine, which is found in MG1655, RNA-seq analyses (GEO # GSE313173) were performed comparing the transcriptomes of LF82 WT vs*.* LF82 β1263A (*E. coli* MG1655 genomic positions 4185030 → 4185032, GCA in MG1655 and GAA in LF82). However, in this case, there were no significantly upregulated or downregulated genes (−1 < Log2 Fold Change > 1, adjusted *P*-value < 0.05), suggesting that by itself, this substitution does not influence gene expression. Thus, the effect of the *rpoB* SNP and whether its presence correlates with LF82 biology is unclear.

## SUMMARY

Although hundreds of studies have sought to determine the etiologies of IBD, the factors that initiate the onset and continuation of CD and UC have so far escaped deep understanding. However, dysbiosis of the gut microbiome and AIEC certainly contribute to disease progression and perhaps to the cause of the disease. The identification of an SNP within the *rpoD* gene of the AIEC LF82 and the ability of this variant to directly increase transcription initiation at specific promoters suggests a molecular mechanism for how this particular pathobiont might gain an advantage within the CD gut. Although the RpoD D445V substitution is exceedingly rare, a valine is also found at the equivalent position in the RpoD sequence of *Fusobacterium varium* (EES64999.2). Interestingly, previous work has found *F. varium* in the mucosal microbiota of patients with UC (116), suggesting that RpoD V445 in this organism might also play a role in this IBD.

The association of RpoD V445 with the upregulation of genes associated with the LF82 lifestyle suggests that the investigation of other SNPs may lead to more understanding about how other AIEC pathobionts function. In particular, the RpoD variant reveals how a single mutation within a highly conserved gene can lead to various functional changes that have the potential to transform a commensal into a pathobiont. Furthermore, this finding demonstrates how pathogenic-like properties of an AIEC may arise from mutations of conserved genes needed for normal functioning, rather than mutations within genes that have been associated with pathogenic properties or the acquisition of pathogenicity islands. The emergence of phenotypes in this manner appears to be an underappreciated process in mechanisms that promote pathogenesis and host interaction.

Finally, it is important to note that while the discovery of the *rpoD* SNP is exciting, it is specific for LF82. It is not found in other AIEC genomes. Although the variant has been found in two other *E. coli* genomes, its potential link to inflammatory diseases in these genetic backgrounds is not known. In addition, as detailed earlier, dozens of genomic differences set various AIEC apart from each other and from other *E. coli*. Consequently, there must be myriad pathways for achieving the similar phenotypes exhibited by these pathobionts. Future work will be needed to narrow down how these pathways are regulated and how SNPs in various genes can manipulate pathways similarly.
